# Irrigation increases and stabilizes mosquito populations and increases West Nile virus incidence

**DOI:** 10.1038/s41598-024-70592-3

**Published:** 2024-08-28

**Authors:** Tony J. Kovach, A. Marm Kilpatrick

**Affiliations:** Department of Ecology and Evolutionary Biology, University of California Santa Cruz, Santa Cruz, CA USA

**Keywords:** Irrigation, Vector-borne disease, Mosquitoes, Climate, Land use, Epidemiology, Ecological epidemiology, Viral infection

## Abstract

Humans have greatly altered earth’s terrestrial water cycle with the majority of fresh water being used for agriculture. Irrigation changes spatial and temporal water availability and alters mosquito abundance and phenology. Previous studies evaluating the effect of irrigation on mosquito abundance and mosquito-borne disease have shown inconsistent results and little is known about the effect of irrigation on variability in mosquito abundance. We examined the effect of irrigation, climate and land cover on mosquito abundance and human West Nile virus (WNV) disease cases across California. Irrigation made up nearly a third of total water inputs, and exceeded precipitation in some regions. Abundance of two key vectors of several arboviruses, including WNV, *Culex tarsalis* and the *Culex pipiens* complex, increased 17–21-fold with irrigation. Irrigation reduced seasonal variability in *C. tarsalis* abundance by 36.1%. Human WNV incidence increased with irrigation, which explained more than a third (34.2%) of the variation in WNV incidence among California counties. These results suggest that irrigation can increase and decouple mosquito populations from natural precipitation variability, resulting in sustained and increased disease burdens. Shifts in precipitation due to climate change are likely to result in increased irrigation in many arid regions which could increase mosquito populations and disease.

## Introduction

Humans have altered many of the Earth’s natural biogeochemical processes, including the terrestrial water cycle^1–3^. The majority of fresh water is used for growing irrigated crops^4–7^, and climate change is predicted to further increase conversion of rain-fed agriculture to irrigation-based systems^8,9^. Irrigation increases and stabilizes agricultural yields by buffering crops against natural variability associated with fluctuations in precipitation, and in doing so alters the timing and spatial availability of water across the landscape^10,11^. Agriculture has previously been associated with increased mosquito abundance^12–15^ and higher rates of human disease including malaria^16,17^, Japanese encephalitis^18^, lymphatic filiaris^19^, Rift Valley fever^20^, and West Nile virus encephalitis^21–23^. However, the effects of irrigation itself are mixed and context dependent, with some studies showing an increase in mosquito abundance with irrigation (especially in rice fields^17,24–26^) while others showed a decrease^27,28^, sometimes due to mosquito predators benefiting more from irrigation than mosquitoes. In addition, less is known about the effects of irrigation on variability in disease risk, despite the importance of irrigation in many arid regions^29^. Examining the effect of irrigation on mosquito populations and vector-borne disease requires simultaneously examining the effects of land use and climate^30–32^.

California is one of the largest irrigated agricultural regions in the world^33,34^, and irrigation during dry months (April–October) is hypothesized to both increase mosquito abundance and decrease its seasonal variability^35,36^. Agriculture has been associated with increased abundance of a key mosquito vector of many viruses, *Culex tarsalis*^14,23,37,38^ and higher human WNV disease incidence^22,23,31,39,40^, and *Culex tarsalis* abundance sometimes increases with irrigation water releases^14,15,37^. More generally, irrigation enables several species of mosquitoes to inhabit otherwise inhospitably dry regions^35,41,42^. These effects of irrigation likely contribute to the effects of land use on mosquito abundance and vector borne disease in California.

While these studies suggest that mosquito populations and mosquito-borne disease often increase with water availability, the effects of rainfall on mosquito populations and WNV human disease rates in the western USA have been mixed. Some studies have found positive associations between increased rainfall and *C. tarsalis* mosquito populations^41,43^ while others have found no effect^44^. WNV incidence has been associated with both increased and decreased rainfall^40,45^, decreased rainfall and drought^46–48^, and moderate rainfall amounts^49^. These inconsistent relationships with rainfall may result from unmeasured effects of irrigation or land cover that affect mosquito larval habitat.

Our goal was to examine the effect of irrigation on the magnitude and variability in mosquito populations and WNV human disease incidence across California. First, we quantified water amounts coming from irrigation and precipitation across space and time. We then examined irrigation, climate, and land cover data as predictors of mosquito abundance, seasonal variability in mosquito populations, and WNV human disease incidence. We focused on three key WNV vectors, *C. tarsalis*, *C. erythrothorax*, and *C. pipiens* complex (*C. pipiens and C. quinquefasciatus*). We hypothesized that irrigation would increase mosquito abundance and WNV incidence and reduce seasonal variability in mosquito populations, likely by stabilizing water availability for larval mosquito development^50^.

## Results

**Fig. 1:**
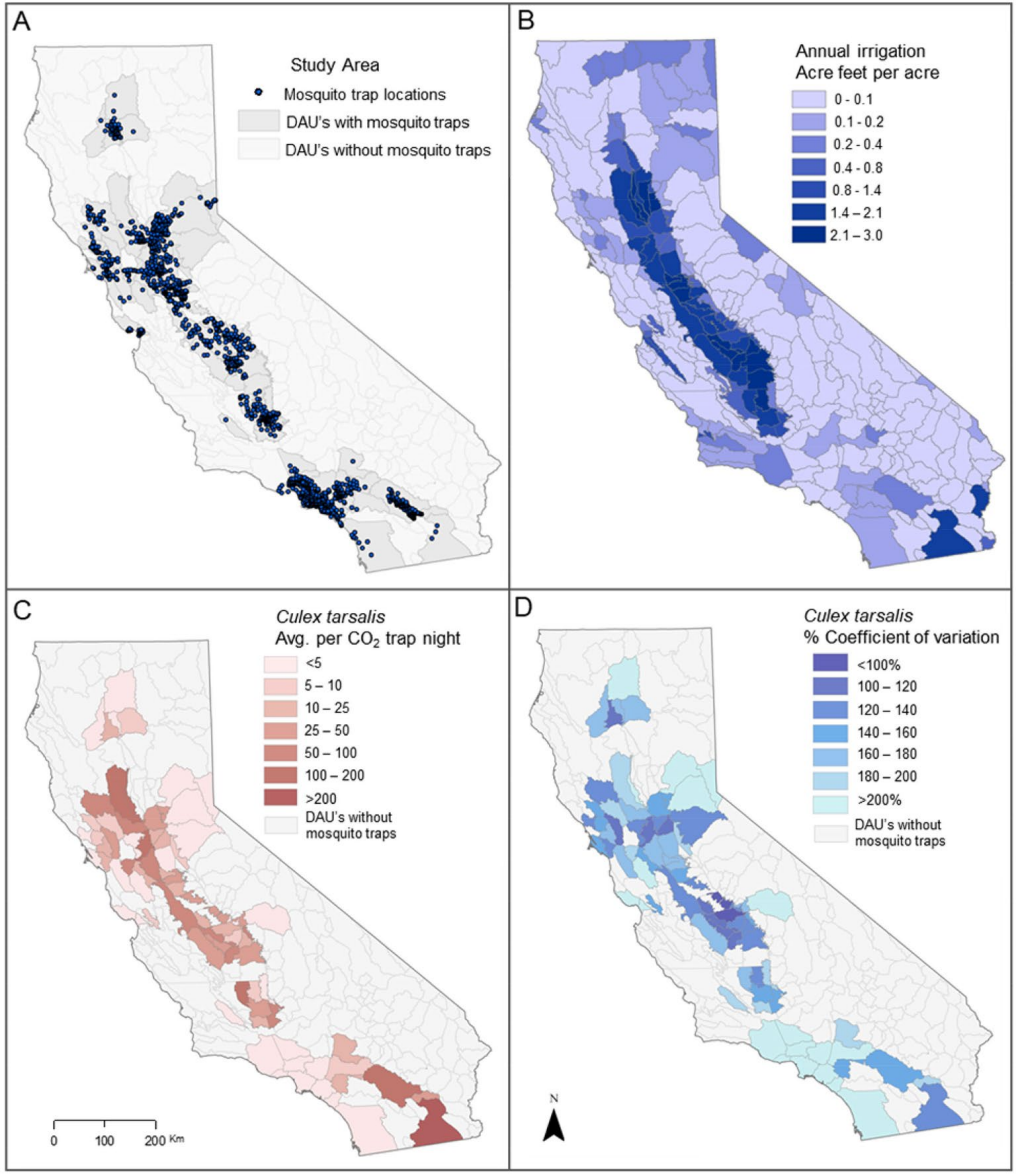
Mosquito trap locations, temperature, precipitation and irrigation shown across California for 1998–2010. (**A**) mosquito CO_2_ trap locations (n = 1336) and detailed analysis units (DAU) with mosquito traps (n = 78). (**B**) Average annual irrigation (typically from April-Oct) for each DAU obtained from California department of water resources^51^. (**C**) Average *C. tarsalis* per CO_2_ trap-night in each detailed analysis unit {average across sites [average across years (average per trap site-year)]}. (**D**) Average coefficient of variation in *C. tarsalis* abundance in each DAU (average across sites (average across years (CV per trap site-year))).

**Fig. 2:**
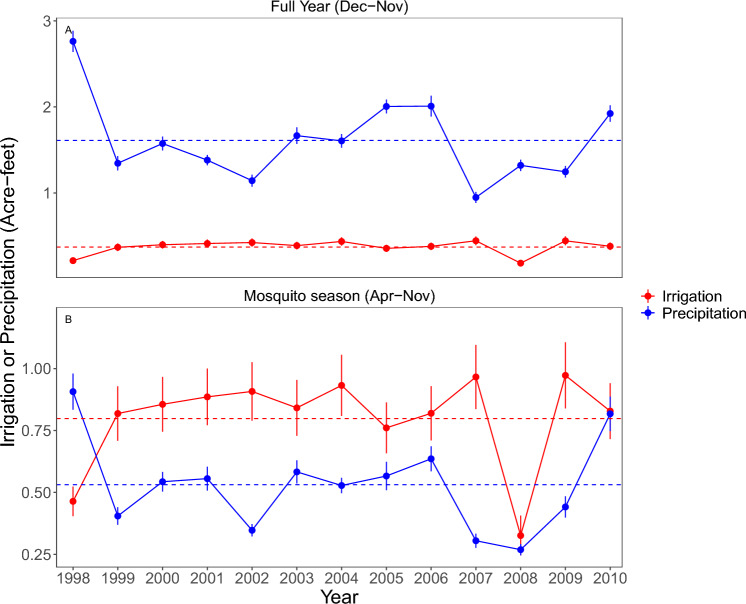
Yearly and warm seasonal precipitation and irrigation. (**A**) Average yearly precipitation (Dec.–Nov., solid black line) and average yearly irrigation (dashed blue line) within study region (n = 78 detailed analysis units). (**B**) Average precipitation during mosquito trapping season (April–November) compared to irrigation within study region. Dashed lines display average across all years.

We examined the influence of irrigation, climate, and land use on spatial and temporal variability in mosquito abundance and across hydrological detailed analysis units (DAU) in California. *Culex tarsalis* abundance varied over four orders of magnitude across California (0.03–555 mosquitoes per trap/night), and seasonal variability of *C. tarsalis* populations (measured using percent coefficient of variation) varied five-fold (76–412%) (Fig. 1). Irrigation was also spatially variable (0–2.97 Acre-feet per acre; Fig. 1), with irrigation making up 18.7% of total annual water (irrigation + precipitation) across the state and 30.9% of total water inputs in the 78 DAUs where there were mosquito data (Fig. 2a). Water from irrigation exceeded that from precipitation during the seasonally dry mosquito trapping season for all but one of 13 years of the study period (Fig. 2b).

**Fig. 3:**
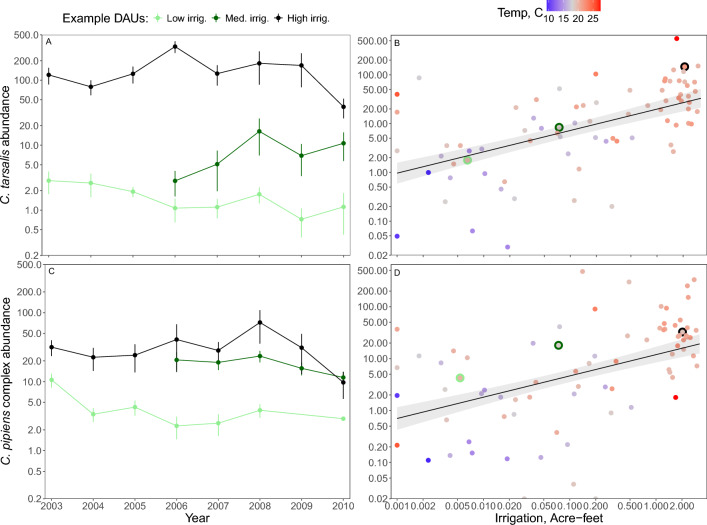
Irrigation and mosquito abundance. Example time series of average annual (**A**) *C. tarsalis* and (**C**) *C. pipiens* complex abundance (± SE) on a log_10_ scale for three DAUs differing in irrigation amounts.. Average (**B**) *C. tarsalis* and (**D**) *C. pipiens* complex abundance per CO_2_ trap-night on a log_10_ scale in each DAU plotted against average irrigation amount (A﻿cre-feet/acre) in each DAU, both averaged across years. Univariate general least squares model including spatial autocorrelation: (**B**) Log_10_ *C. tarsalis* abundance = 1.30 + 0.44 (± SE = 0.089) *log_10_(Irrigation + 0.001); R^2^ = 31%, N = 78; *P* < 0.0001; (**D**) Log_10_ *C. pipiens* complex abundance = 1.08 + 0.41 (± SE = 0.092) *log_10_(Irrigation + 0.001); R^2^ = 24.9%, N = 78; *P* < 0.0001. Multiple regression models with land use and climate predictors are in Table S3, S4. The three larger points with light green, dark green and black outlines in (**B**) and (**D**) show example DAUs shown in panels (**A**) and (**C**), respectively.

In univariate analyses, *C. tarsalis* abundance increased with irrigation (Fig. 3A,B; Table S2), temperature, wetland cover, and decreased with precipitation (Table S2). In the multiple regression model *C. tarsalis* abundance increased with irrigation, average temperatures, wetland landcover, and decreased with developed land cover, but was not significantly correlated with precipitation or open water cover (Table S3). In the univariate model, *C. tarsalis* mosquito abundance more than doubled (2.74-fold higher) with each tenfold increase in irrigation, and increased 20.6-fold across the 1000-fold range of irrigation amounts (Fig. 3A,B, Table S2).

In univariate analyses, *C. pipiens complex* mosquito abundance also increased with irrigation (increasing 17.0-fold across the range of irrigation), temperature developed land cover, and decreased with precipitation and open water (Fig. 3C, D; Table S4). In the multiple regression model, *C. pipiens complex* mosquito abundance increased with irrigation and developed land cover, and decreased with open water, but was uncorrelated with other climate and land use predictors (Table S5). Finally, *C. erythrothorax* abundance decreased with irrigation and precipitation, and increased with wetland land cover (Table S6).

**Fig. 4:**
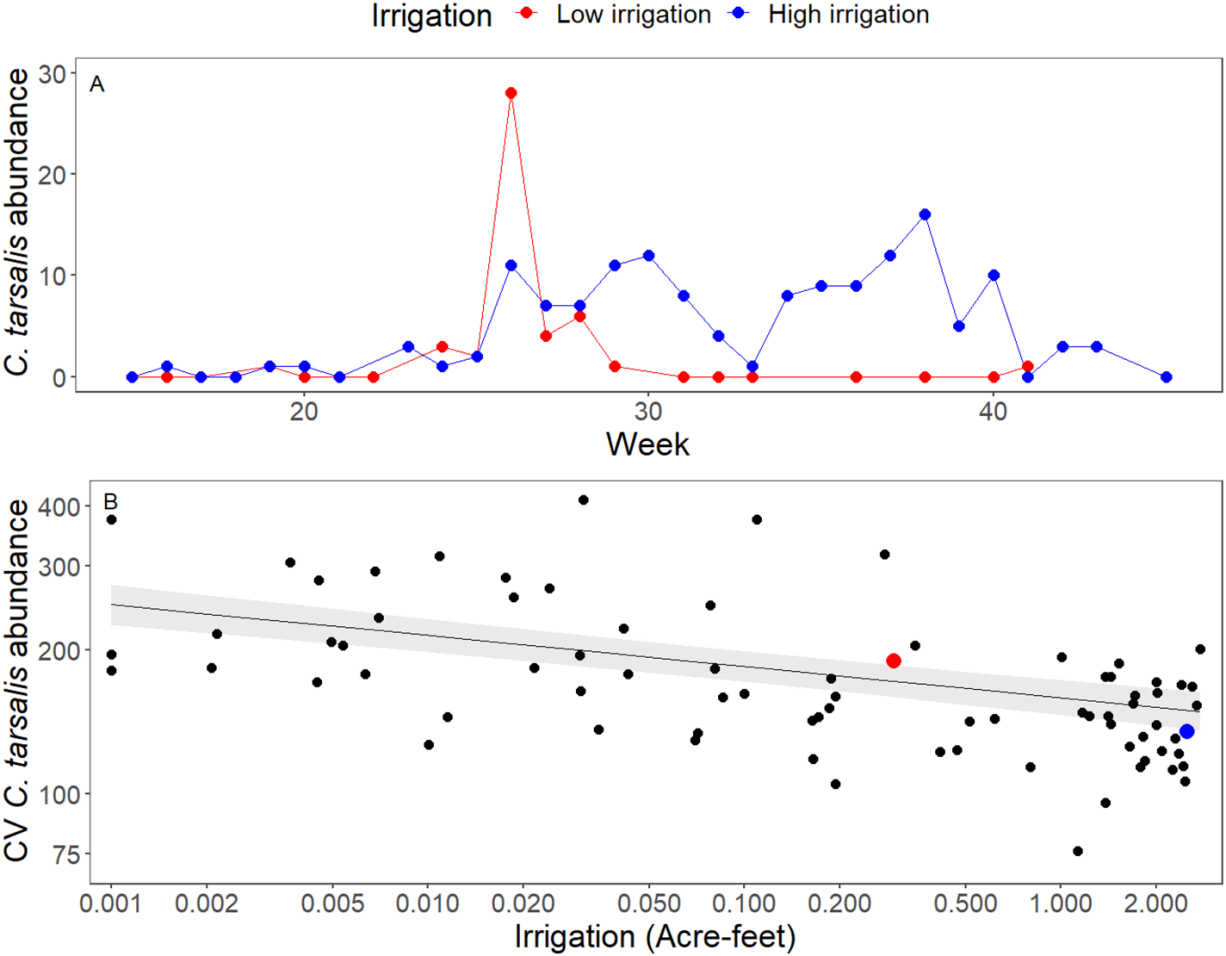
Irrigation effects on seasonal variation in mosquito abundance. (**A**) Example time series for 2010 showing *C. tarsalis* abundance per CO_2_ trap-night at 2 trap sites located in adjacent DAU’s which differed in irrigation amounts. (**B**) Average coefficient of variation in *C. tarsalis* abundance on a log_10_ scale in each DAU {average across sites [average across years (CV per trap site-year)]} plotted against average irrigation amount (Acre-feet/acre) on a log_10_ scale in each DAU. Univariate general least squares model including spatial autocorrelation: Log_10_ CV *C. tarsalis* abundance = 2.20—0.065 (± SE = 0.016) * log_10_(Irrigation + 0.001); R^2^ = 24%; N = 78; *P *< 0.0001). The full model with land use and climate predictors is shown in Table S5. The two colored points show the same DAUs as the time series in panel A.

Seasonal variability in *C. tarsalis* abundance across DAUs decreased by 36.1% across the range of irrigation observed (Fig. 4) but was not significantly correlated with climate or land cover (Table S7). The decrease in variability was due primarily to sustained periods of higher abundance in high-irrigation areas, whereas in low-irrigation areas there was usually a single sharp peak in abundance (Fig. 4A). Seasonal variation in in *C. pipiens complex* mosquito abundance was marginally negatively correlated with irrigation but was not correlated with any other predictors (Table S8). Seasonal variation in *C. erythrothorax* abundance decreased with greater wetland land cover but was uncorrelated with irrigation or other variables (Table S9).

**Fig. 5:**
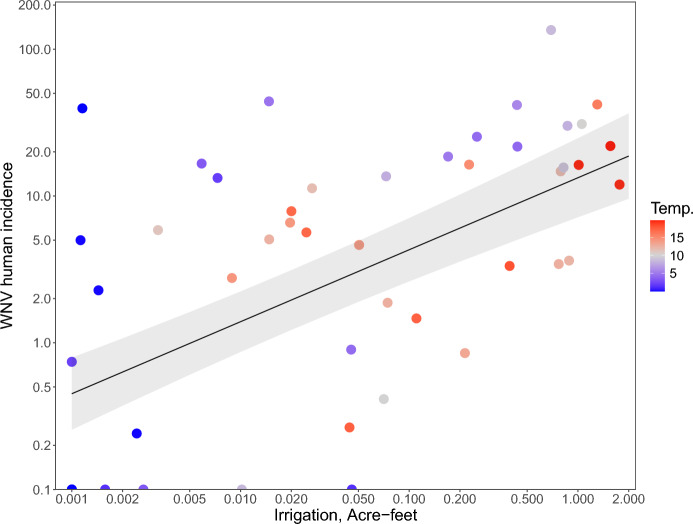
Human WNV disease incidence increases with Irrigation (both on a log_10_ scale) in California counties. Univariate general least squares model including spatial autocorrelation: Log_10_ (Human WNV incidence + 0.1) = 1.12 + 0.49 (± SE = 0.10) * log_10_(Irrigation + 0.001); R^2^ = 34.2%; N = 47; *P* < 0.0001. Point color shows the mean temperature in degrees Celsius, which was not significant in the multiple regression model with land use and climate predictors (Table S11). WNV human disease incidence is average number of WNV cases per year/100,000 people for 2004–2010.

Finally, we examined the effect of irrigation, climate and land use on human WNV disease incidence among California counties. WNV incidence between 2004 and 2010 increased with irrigation, precipitation, temperature, developed land cover, wetland land cover and open water cover in univariate relationships (Table S10). However, the strongest relationship was with irrigation and incidence was not significantly correlated with any climate (precipitation or temperature) or land cover variable once irrigation was accounted for (Fig. 5; Table S11). Irrigation alone explained 34.2% of the variation in human WNV disease incidence among California counties (Fig. 5).

## Discussion

We found that irrigation was correlated with increased abundance and reduced seasonal variability of the two most important mosquito vectors in California, *C. tarsalis* and *C. pipiens* complex, and with increased human WNV incidence. The reduced variability in *C. tarsalis* populations resulted from irrigation increasing periods of elevated abundance, which is consistent with previously observed multi-modal seasonal peaks of *C. tarsalis* abundance in regions of California with heavy irrigation inputs^35^. These relationships suggest that irrigation can partially decouple mosquito abundance, for some species, from natural precipitation patterns^29^ and result in elevated and sustained transmission of mosquito-borne disease. The effect of irrigation on both *C. tarsalis* and *C. pipiens* abundance likely results from irrigation increasing larval habitat for these species^50^.

Irrigation has also been found to alter seasonal variation in mosquito abundance and transmission of some other mosquito-borne diseases, with the best studied disease being malaria in Africa. There, as we found in California, irrigation both increased *Anopheles* mosquito abundance and dampened seasonal variability, with the strongest effects occurring in dry regions^16,52,53^. Irrigation in drier months enabled year-round malaria transmission^29,52,54^. As a result, as we found for mosquitoes in California, human malarial transmission in heavily irrigated regions was no longer tightly coupled with natural precipitation patterns^29^.

We found correlations between mosquito abundance and land use that match previous associations, but climate relationships were somewhat unexpected. Both *C. tarsalis* and *C. erythrothorax* abundance increased with wetland area and *C. pipiens* complex mosquitoes increased with developed land, as expected from other studies^15,23,55–60^. However, while *C. tarsalis* and *C. pipiens* complex mosquito abundance increased with temperature in univariate analyses, the significant correlation was not present in multiple regression analyses, suggesting the correlation with temperature for this group of mosquitoes may not have been causal. In addition, there was no evidence that the relationship between *C. tarsalis* abundance and temperature, was non-linear, as might be expected based on the increased developmental rate and decreased survival rate with temperature^61–63^. Finally, only *C. erythrothorax* abundance was correlated with precipitation, and abundance decreased with increasing precipitation and irrigation, which is somewhat counterintuitive. None of the climate predicators were correlated with seasonal variability in abundance of any of the three species. Overall, the data from California suggest that spatial variation in mosquito abundance and seasonal variability at the scale we examined is more influenced by irrigation and land use than climate.

Three notable limitations affected this study. First, irrigation data was only available at the DAU spatial scale and annual temporal scale. Finer scale analyses may have uncovered even stronger or more detailed relationships, especially for the short-term effects of irrigation on mosquito abundance within a year, which are critical for reducing mosquito larval habitat^64^. The effects of climate variables may have been more apparent at other spatial or temporal scales. Second, we were unable to obtain data after 2010, so we weren’t able to examine the impact of multiple severe droughts and floods on mosquitoes and WNV that occurred after 2010. Similarly, it’s possible the effects of irrigation on WNV might differ between the initial years after introduction (2004–2010) and the most recent decade. Third, we were not able to incorporate vector control efforts which may have reduced mosquito abundance. Despite these limitations, the strength of the relationships we found between irrigation and mosquito abundance, variability, and WNV suggest that irrigation likely plays an important role in transmission of mosquito borne pathogens in this region.

More broadly, the patterns we observed suggest that manipulations to the water cycle increase the magnitude and reduce the variability of multiple mosquito species. Improvements in irrigation water delivery systems (e.g. fixing leaky irrigation canals), irrigation methods (e.g. sub-surface drip irrigation), irrigation pricing schemes (farmers paying for actual amounts of water used rather than supply), and potentially, intermittent irrigation^64^, might reduce the impacts of irrigation on mosquito populations by limiting potential breeding sites^53,65,66^. A key challenge in the coming decades will be finding ways to use irrigation to maintain food security, without increasing mosquito populations and disease risk.

## Methods

### Irrigation data

We obtained annual irrigation estimates (“Applied water”, Acre-feet per acre) for each “detailed analysis unit” (DAU) in California from 1998–2010 from the California department of water resources^51^ (2010 was the last year we were able to obtain data). DAUs are sub-watershed scale areas and are the smallest hydrologic study area used for the analysis of water supply and use by the California Water Plan^67^. These annual irrigation estimates for each DAU are based on the growing areas of 20 agricultural crop categories, each with specific crop coefficients corresponding to estimated annual water use^51^. In each DAU, we multiplied the growing area of each crop by estimates of the irrigation water for growing each crop to calculate annual estimates of total irrigated water for each DAU.

### Mosquito abundance data

We obtained mosquito trapping data collected by vector control districts across California via the CALSURV vector-borne disease surveillance system^68^. These public health agencies focus mosquito trapping efforts near human population centers, in regions with urban and agricultural land use. We used CO_2_ trapping data from the months April through November over a 13-year period during which irrigation data was also available (1998–2010). We included trap sites with at least 10 visits per year (n = 1336 sites), for a total of ~ 80,000 unique site visits in 78 DAU. We calculated average *C. tarsalis* abundance estimates (mosquitoes per CO_2_ trap-night) for each DAU by first by averaging across each year for each site (site-year average), then averaging across sites within an DAU (DAU-year), and then averaging all years. We quantified seasonal variability in *C. tarsalis* abundance estimates for each DAU by first by calculating the percent coefficient of variation (CV = 100*sd/mean) for each site-year, and then averaged these CV values across sites and then averaged them across years.

### Temperature and precipitation data

We obtained spatial estimates of average monthly temperature and precipitation data across California at 4 km resolution^36^. We used ArcGIS to calculate average annual estimates of temperature and precipitation for each DAU across California (n = 278), including the 78 detailed analysis regions with mosquito trapping data. We calculated the average annual precipitation (Dec-Nov, in mm)^41^ and average temperature (°C) during each mosquito trapping season (Apr–Nov) in each DAU for the years 1998–2010. To make precipitation and irrigation amounts comparable, we converted precipitation (mm) into volumetric amounts of water per acre (Acre-feet per acre; 304 mm/foot).

### Land cover data

Within each DAU, we summarized percent land cover of several land cover classes that past analyses indicated were important in mosquito abundance (developed areas, wetlands, open water;^23,31,69^) using datasets available from 2010 USDA National Agricultural Statistics Service (30 m resolution;^70^). Developed areas included a combination of low, medium, and high intensity, as well as open space developed. Wetland areas included a combination of woody and herbaceous wetlands. We did not include fraction agricultural land as an independent variable because the irrigation predictor was created using agricultural land use fractions of 20 different crops and was therefore highly correlated with irrigation (r = 0.94, N = 78, *P* < 0.001).

### WNV human disease cases

We compiled reported human cases of WNV for each California county from the Centers for Disease Control and Prevention (CDC) ArboNET program for the years 2004–2010^71^ (2010 was the last year we were able to obtain data). Average human WNV disease incidence was calculated as the mean number of all cases (fever and neuro-invasive cases combined) per year divided by the county’s population, multiplied by 100,000. We converted DAU estimates of irrigation to county estimates based on weighted averages of county area overlapping with DAU areas. Counties with < 75% overlap with DAU areas were discarded.

### Statistical analyses

All statistical analyses were done using the program R (v. 3.5.0) with spatial summaries and maps made using ArcGIS (v. 10.5). We used generalized least squares models in the R package *gls*, with exponential spatial autocorrelation using the DAU centroid, to examine the effects of climate, land cover, and irrigation on the abundance and seasonal variation in mosquito populations, and human WNV disease incidence. We log_10_ transformed predictor and response variables to linearize relationships and increase normality of residuals (Table S1). We examined non-linearity in relationships between response variables and temperature by comparing models with a quadratic temperature term in each analysis to models without it. For all response variables, models with quadratic terms had higher Akaike Information Criteria values so linear models were used. We examined normality with Shapiro-Wilke tests and used R package PiecewiseSEM to calculate R^2^ values for gls models.

### Supplementary Information

Supplementary Information.

## Data Availability

R Code and data to reproduce the figures and analyses is available at: https://github.com/marmkilpatrick/IrrigationMosquitoesWNV.
