# Supplementary material for: Irrigation increases and stabilizes mosquito populations and increases West Nile virus incidence
Source: Sci Rep. 2024 Aug 28;14:19913. doi: 10.1038/s41598-024-70592-3 (PMC11358498; doi:10.1038/s41598-024-70592-3)
Supplement: Supplementary file 1 — Supplementary Information. [file 41598_2024_70592_MOESM1_ESM.docx]

**Supporting Information for:**

**Irrigation increases and stabilizes mosquito populations and increases West Nile virus incidence**

**Tony J. Kovach and A. Marm Kilpatrick**

Department of Ecology and Evolutionary Biology, University of California Santa Cruz, Santa Cruz, CA, USA

* To whom correspondence should be addressed; emails: [tokovach@gmail.com](mailto:tokovach@gmail.com), [akilpatr@ucsc.edu](mailto:akilpatr@ucsc.edu)

**Table S1: List of predictor and response variables used in generalized least squares models along with transformations and descriptions. Each variable is summarized as an average per detailed analysis unit. A few variables had a small number of zero values; a small number less than the smallest observed value was added before log transformation, as noted below.**

| Variables | Transformation | Description |
| --- | --- | --- |
| Temperature | none | Average temperature (Celsius), April-Nov, 1998-2010 |
| Precipitation | log_10_ | Average precipitation (Acre-feet-acre),  Dec-Nov, 1998-2010 |
| Irrigation | log_10_ + 0.001 | Average irrigation (Acre-feet-acre),  Apr-Oct, 1998-2010 |
| Wetland | log_10_ | Percent cover of wetland land cover (woody & herbaceous) |
| Developed | log_10_ | Percent cover of developed land cover (High, medium, low intensity and open space developed) |
| Open Water | log_10_ | Percent cover of open water land cover |
| *C. tarsalis* abundance | log_10_ | CO_2_ trap sites with>=10 visits, averaged per site-year, averaged per DAU-year, averaged per DAU |
| *C. pipiens* complex  abundance | log_10_ + 0.1 | CO_2_ trap sites with>=10 visits, averaged per site-year, averaged per DAU-year, averaged per DAU |
| *C. erythrothorax*  abundance | log_10_ + 0.1 | CO_2_ trap sites with>=10 visits, averaged per site-year, averaged per DAU-year, averaged per DAU |
| *C. tarsalis* CV | log_10_ | CO_2_ trap sites with>=10 visits, For each site-year calculated CV=100*(sd/mean), averaged per DAU-year, averaged per DAU |
| *C. pipiens* complex CV | log_10_ | CO_2_ trap sites with>=10 visits, For each site-year calculated CV=100*(sd/mean), averaged per DAU-year, averaged per DAU |
| *C. erythrothorax* CV | log_10_ | CO_2_ trap sites with>=10 visits, For each site-year calculated CV=100*(sd/mean), averaged per DAU-year, averaged per DAU |
| Human WNV  disease incidence | log_10_ + 0.1 | Average yearly human disease incidence (cases/100,000) by county (2004-2010). |

**Table S2: Results from six univariate Generalized Least Squares models including spatial autocorrelation with exponential correlation structure for log_10_(*C. tarsalis* abundance). For each model the table shows the intercept, slope, SE P-value, and R^2^ for a model with just that predictor and the spatial autocorrelation (N= 78 DAUs).**

| Predictor | Intercept | Slope | SE | P-value | R^2^ |
| --- | --- | --- | --- | --- | --- |
| Log_10_(Irrigation+0.001) | 1.30 | 0.44 | 0.089 | 5.2×10^-06^ | 0.31 |
| Log_10_(Precipitation) | 1.06 | -1.76 | 0.35 | 3.2×10^-06^ | 0.36 |
| Temperature | -3.09 | 0.21 | 0.032 | 6.0×10^-09^ | 0.42 |
| Log_10_(% Wetland) | 1.03 | 0.34 | 0.11 | 0.0025 | 0.11 |
| Log_10_(% Developed) | 0.57 | 0.20 | 0.22 | 0.37 | 0.012 |
| Log_10_(% Open Water) | 0.74 | 0.048 | 0.17 | 0.78 | 0.00086 |

**Table S3: Multiple regression Generalized Least Squares model including spatial autocorrelation with exponential correlation structure for log_10_(*C. tarsalis* abundance) using irrigation, climate, and land cover variables.** **Whole model R^2^ = 57.5%; N = 78.**

| Predictor | Coefficient | SE | t-value | P-value |
| --- | --- | --- | --- | --- |
| Intercept | -1.09 | 0.91 | -1.20 | 0.23 |
| Log_10_(Irrigation+0.001) | 0.20 | 0.078 | 2.56 | 0.013 |
| Log_10_(Precipitation) | -0.45 | 0.35 | -1.28 | 0.21 |
| Temperature | 0.15 | 0.045 | 3.25 | 0.0018 |
| Log_10_(% Wetland) | 0.22 | 0.10 | 2.16 | 0.034 |
| Log_10_(% Developed) | -0.33 | 0.15 | -2.13 | 0.037 |
| Log_10_(% Open Water) | 0.23 | 0.15 | 1.52 | 0.13 |

**Table S4: Results from six univariate Generalized Least Squares models including spatial autocorrelation with exponential correlation structure for log_10_(*C. pipiens complex* abundance + 0.1). *C. pipiens* complex mosquitoes included both *C. pipiens* and *C. quinquefasciatus.* For each model the table shows the intercept, slope, SE P-value, and R^2^ for a model with just that predictor and the spatial autocorrelation (N= 78 DAUs).**

| Predictor | Intercept | Slope | SE | P-value | R^2^ |
| --- | --- | --- | --- | --- | --- |
| Log_10_(Irrigation+0.001) | 1.08 | 0.41 | 0.092 | 2.7×^-05^ | 0.25 |
| Log_10_(Precipitation) | 0.79 | -1.13 | 0.36 | 0.0026 | 0.15 |
| Temperature | -1.74 | 0.13 | 0.038 | 0.0016 | 0.15 |
| Log_10_(% Wetland) | 0.70 | 0.16 | 0.12 | 0.20 | 0.021 |
| Log_10_(% Developed) | -0.0017 | 0.64 | 0.22 | 0.0047 | 0.11 |
| Log_10_(% Open Water) | 0.45 | -0.44 | 0.18 | 0.017 | 0.066 |

**Table S5: Multiple regression Generalized Least Squares model including spatial autocorrelation with exponential correlation structure for log_10_(*C. pipiens complex* abundance + 0.1) using irrigation, climate, and land cover variables. *C. pipiens* complex mosquitoes included both *C. pipiens* and *C. quinquefasciatus.* Whole model R^2^ = 38.0%; N = 78.**

| Predictor | Coefficient | SE | t-value | P-value |
| --- | --- | --- | --- | --- |
| Intercept | 1.33 | 1.20 | 1.11 | 0.27 |
| Log_10_(Irrigation+0.001) | 0.35 | 0.11 | 3.25 | 0.0018 |
| Log_10_(Precipitation) | -0.71 | 0.48 | -1.48 | 0.14 |
| Temperature | -0.039 | 0.059 | -0.65 | 0.52 |
| Log_10_(% Wetland) | -0.041 | 0.13 | -0.33 | 0.75 |
| Log_10_(% Developed) | 0.42 | 0.20 | 2.10 | 0.039 |
| Log_10_(% Open Water) | -0.38 | 0.18 | -2.11 | 0.038 |

**Table S6: Multiple regression Generalized Least Squares model including spatial autocorrelation with exponential correlation structure for log_10_(*C. erythrothorax* abundance + 0.1) using irrigation, climate, and land cover variables. Whole model R^2^ = 33.7%; N = 78.**

| Predictor | Coefficient | SE | t-value | P-value |
| --- | --- | --- | --- | --- |
| Intercept | 0.99 | 1.40 | 0.71 | 0.48 |
| Log_10_(Irrigation+0.001) | -0.36 | 0.12 | -3.03 | 0.0034 |
| Log_10_(Precipitation) | -1.59 | 0.54 | -2.95 | 0.0042 |
| Temperature | -0.058 | 0.069 | -0.84 | 0.40 |
| Log_10_(% Wetland) | 0.58 | 0.15 | 3.77 | 0.0003 |
| Log_10_(% Developed) | 0.41 | 0.23 | 1.77 | 0.082 |
| Log_10_(% Open Water) | -0.12 | 0.23 | -0.54 | 0.59 |

**Table S7: Multiple regression Generalized Least Squares model including spatial autocorrelation with exponential correlation structure for the Coefficient of Variation (CV) of *C. tarsalis* abundance within each mosquito trapping season, using climate, irrigation and land cover variables.** **Whole** **model R^2^ = 33.4%; N = 78.**

| Predictor | Coefficient | SE | t-value | P-value |
| --- | --- | --- | --- | --- |
| Intercept | 2.49 | 0.21 | 12.03 | <0.0001 |
| Log_10_(Irrigation+0.001) | -0.041 | 0.020 | -2.06 | 0.043 |
| Log_10_(Precipitation) | 0.075 | 0.11 | 0.70 | 0.49 |
| Temperature | -0.015 | 0.010 | -1.47 | 0.15 |
| Log_10_(% Wetland) | 0.0012 | 0.018 | 0.07 | 0.95 |
| Log_10_(% Developed) | -0.0097 | 0.032 | -0.30 | 0.76 |
| Log_10_(% Open Water) | -0.027 | 0.024 | -1.12 | 0.27 |

**Table S8: Multiple regression Generalized Least Squares model including spatial autocorrelation with exponential correlation structure for the Coefficient of Variation (CV) of *C. pipiens* complex mosquitoes within each mosquito trapping season, using climate, irrigation and land cover variables. *C. pipiens* complex combines both *C. pipiens* and *C. quinquefasciatus*. Whole model R^2^ = 10.0%; N = 76.**

| Predictor | Coefficient | SE | t-value | P-value |
| --- | --- | --- | --- | --- |
| Intercept | 2.44 | 0.30 | 8.20 | <0.0001 |
| Log_10_(Irrigation+0.001) | -0.054 | 0.028 | -1.89 | 0.063 |
| Log_10_(Precipitation) | -0.13 | 0.15 | -0.85 | 0.40 |
| Temperature | -0.0059 | 0.014 | -0.41 | 0.68 |
| Log_10_(% Wetland) | 0.040 | 0.025 | 1.57 | 0.12 |
| Log_10_(% Developed) | -0.044 | 0.046 | -0.96 | 0.34 |
| Log_10_(% Open Water) | 0.0099 | 0.036 | 0.27 | 0.79 |

**Table S9: Multiple regression Generalized Least Squares model including spatial autocorrelation with exponential correlation structure for the Coefficient of Variation (CV) of *C. erythrothorax* abundance within each mosquito trapping season, using climate, irrigation and land cover variables. Whole model R^2^ = 11.0%; N = 64.**

| Predictor | Coefficient | SE | t-value | P-value |
| --- | --- | --- | --- | --- |
| Intercept | 2.03 | 0.32 | 6.39 | <0.0001 |
| Log_10_(Irrigation+0.001) | 0.014 | 0.026 | 0.56 | 0.58 |
| Log_10_(Precipitation) | 0.22 | 0.14 | 1.55 | 0.13 |
| Temperature | 0.019 | 0.015 | 1.23 | 0.22 |
| Log_10_(% Wetland) | -0.069 | 0.034 | -2.06 | 0.045 |
| Log_10_(% Developed) | 0.012 | 0.045 | 0.28 | 0.78 |
| Log_10_(% Open Water) | 0.037 | 0.053 | 0.70 | 0.49 |

**Table S10: Results from six univariate Generalized Least Squares models including spatial autocorrelation with exponential correlation structure for human WNV disease incidence (average number of WNV cases per year/100,000 people for 2004-2010) within California counties. For each model, N= 47 counties. For each model the table shows the intercept, slope, SE P-value, and R^2^ for a model with just that predictor and the spatial autocorrelation.**

| Predictor | Intercept | Slope | SE | P-value | R^2^ |
| --- | --- | --- | --- | --- | --- |
| Log_10_(Irrigation+0.001) | 1.12 | 0.49 | 0.10 | 0.000026 | 34.2% |
| Log_10_(Precipitation) | 0.43 | 0.24 | 0.086 | 0.0076 | 11.9% |
| Temperature | -0.018 | 0.045 | 0.021 | 0.042 | 10.4% |
| Log_10_(% Wetland) | 0.76 | 0.26 | 0.066 | 0.00032 | 24.1% |
| Log_10_(% Developed) | 0.30 | 0.26 | 0.075 | 0.0011 | 19.0% |
| Log_10_(% Open Water) | 0.47 | 0.22 | 0.083 | 0.013 | 11.3% |

**Table S11: Multiple regression Generalized Least Squares model including spatial autocorrelation with exponential correlation structure (estimated range: 0.61) for human WNV disease incidence (average number of WNV cases per year/100,000 people for 2004-2010) within (N= 47) California counties, using climate, irrigation and land cover predictors. The whole model R^2^ was 37.1%.**

| Predictor | Coefficient | SE | t-value | P-value |
| --- | --- | --- | --- | --- |
| Intercept | 0.84 | 0.54 | 1.57 | 0.12 |
| Log_10_(Irrigation+0.001) | 0.44 | 0.19 | 2.36 | 0.023 |
| Log_10_(Precipitation) | 0.40 | 0.41 | 0.98 | 0.33 |
| Temperature | 0.0019 | 0.028 | 0.069 | 0.95 |
| Log_10_(% Wetland) | -0.013 | 0.20 | -0.063 | 0.95 |
| Log_10_(% Developed) | 0.16 | 0.23 | 0.67 | 0.51 |
| Log_10_(% Open Water) | -0.47 | 0.37 | -1.26 | 0.21 |
